# Designing an evidence-based mobile health decision aid for the management of hypertensive disorders of pregnancy (HDP) in low-resourced settings

**DOI:** 10.1186/1472-6963-14-S2-P93

**Published:** 2014-07-07

**Authors:** Beth Payne, Dustin Dunsmuir, David Hall, Joanne Lim, Rozina Sikandar, Laura Magee, Rahat Qureshi, Mark Ansermino, Peter von Dadelszen

**Affiliations:** 1 Department of Obstetrics and Gynaecology, University of British Columbia, Vancouver, BC, Canada; 2 Department of Anesthesiology, University of British Columbia, Vancouver, BC, Canada; 3 Department of Obstetrics and Gynaecology, Stellenbosch University, Tygerberg, Western Cape, South Africa; 4 Department of Obstetrics and Gynaecology, Aga Khan University, Karachi, Pakistan

## Background

Pre-eclampsia is one of the leading causes of maternal death and morbidity in low-resourced countries due to delays in case identification and a shortage of health workers trained to manage the disorder. The objective of the PIERS on the Move (POM) project was to provide mid-level health workers with an evidence-based and low-cost decision aid to improve diagnosis and management of pre-eclampsia, to improve outcomes.

## Materials and methods

The decision algorithm used in the POM application was designed through iterative review by study working group members and incorporated several risk thresholds as triggers for recommendations of interventions. The WHO recommendations for treatment and management of pre-eclampsia and eclampsia were also used to define the treatment recommendations made.

The POM application was used to collect a prospective cohort of women admitted to hospital with an HDP in South Africa and Pakistan. The accuracy of the decision algorithm overall was assessed based on the algorithm’s ability to correctly identify high-risk cases (women who went on to suffer an adverse maternal outcome). During this study, recommendations for care generated by POM were blinded to the clinical and research staff.

## Results

Between 1 January 2011 and 31 March 2012, 617 women were recruited to the study in Pakistan, while 235 women were recruited in South Africa between 1 November 2012 and 31 December 2013, creating a total cohort of 852 women of whom 119 (14.0%) experienced one or more component of a composite adverse maternal outcome within 48 hours of admission. During the study, the research staff reported high usability and acceptance of the tool in the clinical setting.

When the POM decision algorithm was applied to the study cohort, 339 women were identified as high-risk requiring further treatment. Of these, 89 (26.3%) suffered an adverse maternal outcome within 48 hours of admission to hospital. Use of the POM decision aid correctly identified 74.8% of high-risk women, with false positive rate of 29.3% and overall accuracy of 67.1%.

## Conclusions

The POM decision aid showed moderate accuracy in the study cohort, was designed with user input and is acceptable by health workers in low-resourced settings. The true effect of the POM tool on maternal outcomes needs to be assessed in an implementation study.

